# In-host microevolution of *Aspergillus fumigatus*: A phenotypic and genotypic analysis

**DOI:** 10.1016/j.fgb.2018.02.003

**Published:** 2018-04

**Authors:** Eloise Ballard, Willem J.G. Melchers, Jan Zoll, Alistair J.P. Brown, Paul E. Verweij, Adilia Warris

**Affiliations:** a Medical Research Council Centre for Medical Mycology at the University of Aberdeen, Aberdeen Fungal Group, Institute of Medical Sciences, Aberdeen, UK; b Department of Medical Microbiology, Radboud University Medical Centre, Nijmegen, The Netherlands; c Centre of Expertise in Mycology, Radboudumc/CWZ, Nijmegen, The Netherlands

**Keywords:** *Aspergillus fumigatus*, In-host microevolution, Azole-resistance, Fungal growth, Whole genome sequencing

## Abstract

In order to survive, *Aspergillus fumigatus* must adapt to specific niche environments. Adaptation to the human host includes modifications facilitating persistent colonisation and the development of azole resistance. The aim of this study is to advance understanding of the genetic and physiological adaptation of *A. fumigatus* in patients during infection and treatment. Thirteen *A. fumigatus* strains were isolated from a single chronic granulomatous disease patient suffering from persistent and recurrent invasive aspergillosis over a period of 2 years. All strains had identical microsatellite genotypes and were considered isogenic. Whole genome comparisons identified 248 non-synonymous single nucleotide polymorphisms. These non-synonymous mutations have potential to play a role in in-host adaptation. The first 2 strains isolated were azole susceptible, whereas later isolates were itraconazole, voriconazole and/or posaconazole resistant. Growth assays in the presence and absence of various antifungal stressors highlighted minor changes in growth rate and stress resistance, with exception of one isolate showing a significant growth defect. Poor conidiation was observed in later isolates. In certain drug resistant isolates conidiation was restored in the presence of itraconazole. Differences in virulence were observed as demonstrated in a *Galleria mellonella* infection model*.* We conclude that the microevolution of *A. fumigatus* in this patient has driven the emergence of both Cyp51A-independent and Cyp51A-dependent, azole resistance mechanisms, and additional phenotypes that are likely to have promoted fungal persistence.

## Highlights

• Description of in-host adaptation of *A. fumigatus* promoting fungal persistence.
• Significant in-host microevolution was identified in 13 consecutive *A. fumigatus* isolates from a single patient.
• Emerging azole resistance, fungal growth, conidiation and virulence differences were observed.
• 248 non-synonymous single nucleotide polymorphisms developed throughout series.

## 1 Introduction

*Aspergillus fumigatus* is a ubiquitous saprophytic fungus. Its ecological niche is soil, where it plays a key role in carbon and nitrogen recycling by degradation of organic biomass (O’Brien et al., 2005, Tedersoo et al., 2014). Various characteristics enable *A. fumigatus* to survive in this harsh environment, including rapid germination, growth at higher temperatures and nutritional metabolic flexibility (Mullins et al., 1976). This versatility enables *A. fumigatus* to be a successful pathogen in human, animal and plant populations (Armstrong-James et al., 2016, Fisher et al., 2012).

In humans, *A. fumigatus* is the causative agent of aspergillosis, which ranges from allergic syndromes to life-threatening invasive aspergillosis (Latgé, 2001, Warris, 2014). Azole antifungal agents hold great importance in the treatment of aspergillosis, as they are the only orally available anti-*Aspergillus* agents (Patterson et al., 2016, Peyton et al., 2015, Warris, 2014). The primary target of azoles is cytochrome P450 14α-sterol demethylase (Cyp51A), which catalyses the demethylation of ergosterol precursors in the ergosterol biosynthetic pathway (Bosschef et al., 1995, Ghannoum and Rice, 1999, Shapiro et al., 2011). Azoles competitively inhibit Cyp51A by binding to the haem active site (Gollapudy et al., 2004, Xiao et al., 2004).

In order to survive and thrive in-host, *A. fumigatus* must adapt to specific niche environments (Verweij et al., 2016). Genetic adaptation can be defined as the acquisition of heritable modifications, via either spontaneous mutation or recombination, which enable survival and reproduction in the environment (Schoustra et al., 2005). Examples of adaptation include adaptation to enable persistent infection and azole resistance development (Fedorova et al., 2008, Hagiwara et al., 2014, Kano et al., 2015, Valsecchi et al., 2015, Verweij et al., 2016). The in-host acquisition of resistance has previously been described within aspergillomas in chronic pulmonary aspergillosis (Howard et al., 2013).

Since the first report of itraconazole resistance in *A. fumigatus* in 1997 (Denning et al., 1997), azole resistance is increasingly reported globally. A range of molecular mechanisms conferring azole resistance have been described. Specific non-synonymous point mutations in *cyp51A* have been shown to confer azole resistance by altering the ligand entry channel structure; examples include G54, P216, G138 and M220 (Albarrag et al., 2011, Garcia-Effron et al., 2005, Mann et al., 2003, Mellado et al., 2004, Mellado et al., 2003, Snelders et al., 2010). A tandem repeat of 34 bp in the promoter region of *cyp51A* has also been shown to confer itraconazole resistance by increasing *cyp51A* gene expression in combination with an L98H mutation within *cyp51A* (Mellado et al., 2007, Snelders et al., 2008). In contrast to *cyp51A*-mediated resistance mechanisms, relatively few non *cyp51A*-mediated mechanisms have been described. One example is a P88L substitution in the CCAAT-binding transcription factor complex subunit HapE, which has been shown to confer itraconazole resistance by enhancing *cyp51A* expression (Camps et al. 2012). Overexpression of efflux transporters AtrF and Cdr1B has been associated with azole resistance (Fraczek et al., 2013, Slaven et al., 2002) but further research is required to validate the role of these pumps in azole resistance. Mutation in components of mitochondrial complex I, RamA (farnesyltransferase β-subunit), overexpression of *cyp51B* and deletion of cytochrome b_5_ CybE have also been described to result in azole resistance (Bromley et al., 2016, Buied et al., 2013, Misslinger et al., 2017, Norton et al., 2017).

Cyp51A-mediated resistance mechanisms are not thought to be associated with fitness costs (Lackner et al., 2017, Valsecchi et al., 2015). In contrast, the HapE itraconazole resistance mechanism is associated with a growth defect (Camps et al. 2012). Interestingly, specific azole resistant isolates are hypothesised to be ‘azole addicted’ whereby they exhibit enhanced growth in the presence of azole antifungals (Anderson, 2005, Schoustra et al., 2006).

Studies investigating the dynamics of in-host adaptation and persistent infection are scarce. Here we performed a detailed phenotypic and genotypic analysis of 13 *A. fumigatus* isolates consecutively cultured over a period of 2 years with increasing azole resistance in a chronic granulomatous disease (CGD) patient with chronic and recurrent aspergillosis. Whole genome sequencing was used to assess the genomic dynamics. Phenotypic analysis including growth in liquid and on solid media and conidiation assays were used to investigate physiological adaptation. An invertebrate infection model was used to assess differences in virulence.

## 2 Materials and methods

### 2.1 Origin and characterisation of fungal isolates

The 13 isolates used in this study were cultured from a 36-year-old male diagnosed with X-linked chronic granulomatous disease with severe chronic obstructive pulmonary disease (Gold IV) and allergic bronchopulmonary aspergillosis (Verweij et al., 2016). The patient suffered from 3 episodes of invasive aspergillosis and developed an aspergilloma, which could not be surgically removed due to his poor respiratory condition. The patient was treated prophylactically with interferon-gamma, trimethoprim-sulphamethoxazole and itraconazole. Between June and December 2011, the patient was treated with itraconazole followed by combination therapy consisting of voriconazole and an echinocandin (caspofungin, anidulafungin). Isolate V130-15 was collected on 22/11/11 and isolates V130-14, V130-18 and V130-54 were collected on 25/11/11. Between December 2011 and January 2013 the patient was treated consecutively with liposomal amphotericin B, itraconazole, anidulafungin in combination with voriconazole. Between August and December 2013, the patient was treated with posaconazole monotherapy, followed by combination therapy with micafungin. Despite these efforts, eradication of the fungus was not achieved. Isolates V157-39, V157-40, V157-47, V157-48 and V157-62 were collected on 9/12/13. Isolates V157-59, V157-60 and V157-61 were collected on 12/12/13; and isolate V157-80 was collected on 19/12/13. Unfortunately the patient died from his infection.

The 13 *A. fumigatus* isolates were cultured and morphologically identified as *A. fumigatus* at Radboud University Medical Centre (Verweij et al., 2016). *In vitro* susceptibility testing of the isolates was performed according to the EUCAST broth microdilution reference method (Subcommittee on Antifungal Susceptibility Testing of the ESCMID European Committee for Antimicrobial Susceptibility Testing (EUCAST), 2015). Isolates were tested at a final drug concentration rage of 0.0312–16 mg/L itraconazole (Sigma Aldrich, UK), 0.0312–16 mg/L voriconazole (Pfizer, UK) and 0.0156–8 mg/L posaconazole (Sigma Aldrich, UK). A no growth end point was determined by eye. Short tandem repeat (STR) typing was performed as described previously using microsatellite loci STR*Af* 3A, 3B, 3C, 4A, 4B and 4C (de Valk et al., 2005). Repeat numbers at each loci were determined by PCR and subsequent sequencing.

### 2.2 Conidial suspension preparation

*A. fumigatus* conidia were spread onto diluted Sabouraud dextrose agar in T75 culture flasks (Greiner Bio-One, Germany) and incubated at 37 °C for 7 d. Diluted Sabouraud dextrose agar was selected to promote sporulation. Conidia were harvested *via* immersion in 30 mL phosphate buffered saline (PBS) (Thermo Fisher Scientific, UK) containing 0.05% Tween-80 (Thermo Fisher Scientific, UK). Conidial suspensions were passed through a sterile 40 µm strainer to remove hyphal fragments, washed twice using PBS and then counted using a Neubauer improved haemocytometer (Petrikkou et al., 2001). For all experiments, suspensions were diluted as required in RPMI (RPMI 1640 + Glutamax, Fisher Scientific, UK).

### 2.3 Whole genome sequencing

DNA was extracted from either conidia or mycelium. Conidia were suspended in TE buffer (pH 8, 1% SDS, 2% Triton X100, 100 mM NaCl). The suspension was shaken for 30 min at 70 °C. DNA was extracted using phenol/chloroform extraction and purified using the QIAamp DNA Blood Mini kit (Qiagen, Germany). A fragmented genomic DNA library was prepared using a Nextera XT DNA sample preparation kit (Illumina, USA). Subsequent sequencing was conducted in a paired end 2 × 150 bp mode using an Illumina NextSeq 500 machine (Illumina, USA).

### 2.4 Bioinformatics analysis

Raw reads were quality checked using FastQC (version 0.11.5, Babraham Institute). Reads containing adapter sequences and/or with a Phred score <30 were removed using Trimmomatic (Galaxy version 0.32.3) (Bolger et al., 2014, Giardine et al., 2005). Reads were mapped to the Af293 reference genome (release 31, EnsemblFungi) using the very sensitive local align preset mode in Bowtie2 (Garcia-Alcalde et al., 2012). Mapping quality was assessed using Qualimap (Garcia-Alcalde et al., 2012, Okonechnikov et al., 2015). Single nucleotide polymorphism (SNP) detection was conducted using FreeBayes (Garrison and Marth, 2012). VCFtools vcf-isec was used to assess patterns amongst SNPs and to filter SNPs with a minimum coverage of 5 and a minimum probability of 0.8 (Danecek et al., 2011). EnsemblFungi Variant Effect Predictor was used to assess the impact of non-synonymous SNPs (Flicek et al., 2014). Both synonymous and non-synonymous SNPs were considered for phylogenetic analysis using the SNPhylo pipeline (Lee et al., 2014), which utilises vcftools (Danecek et al., 2011), Phylip (University of Washington, USA) and Muscle (Edgar, 2004) to generate phylogenetic trees by the maximum likelihood method. Integrated Genomics Viewer and Tablet were utilised for visualisation of sequence data (Boyaval et al., 2007, Milne et al., 2013, Thorvaldsdóttir et al., 2013).

### 2.5 Growth assays

#### 2.5.1 Liquid medium

Flat-bottomed 96-well plates (Nunc microwell 96F, Thermo Fischer Scientific, UK) were seeded with 1.9 × 10^5^ conidia in RPMI. Selected wells were supplemented with specific concentrations of posaconazole (POS 0.5–1 mg/L), voriconazole (VORI 1–4 mg/L) or 2.5 mM *tert*-Butyl hydroperoxide (*t*BOOH) (Sigma Aldrich, UK). Plates were incubated at 37 °C for 48 h inside a spectrophotometric plate reader (FLUOstar OPTIMA, BMG Labtech, Germany). Optical density at 450 nm was automatically measured every 20 min with 5 s shaking before every reading. Each condition was performed in triplicate wells and repeated twice. Due to the lipophilic properties of itraconazole (ITR), specific concentrations were unable to be determined in liquid media; solid media assays were consequently used for studying the impact of ITR on growth.

#### 2.5.2 Solid medium

Sabouraud dextrose agar plates were spot-inoculated with 5 × 10^2^ conidia. Selected plates were supplemented with specific concentrations of either ITR (between 1 and 8 mg/L) or 2.5 mM *t*BOOH. Supplements were added to the medium at ∼50 °C before solidification. Plates were incubated at 37 °C for 96 h, colony diameters were measured every 24 h. Each condition was performed in triplicate.

#### 2.5.3 Environmental zinc depletion

*A. fumigatus* conidia were spread onto glucose minimal agar lacking zinc in T75 culture flasks and incubated at 37 °C for 7 d. Conidia were harvested and counted as described. Zinc depletion experiments were performed by spot inoculating 5 × 10^2^ conidia on glucose minimal media in the absence and presence of 1 mM zinc at pH 4.5 and 7.5 (Amich et al., 2010). Plates were incubated at 37 °C for 96 h, colony diameters were measured every 24 h. Each condition was performed in triplicate.

#### 2.5.4 Conidiation quantification

T75 culture flasks containing Sabouraud dextrose agar were inoculated with 1 × 10^5^ conidia and incubated at 37 °C for 7 d. Selected flasks were supplemented with 4 mg/L ITR. Conidial suspensions were prepared and counted as described above. Where sterile hyphae were produced, a 1 cm^3^ section of hyphae was excised using a sterile plastic loop, re-plated and incubated at 37 °C for an additional 7 d. Conidial suspensions were subsequently prepared and counted as described. Each condition was performed in duplicate.

### 2.6 Galleria mellonella virulence assays

Similar sized *G. mellonella* larvae (Livefood Ltd, UK) were selected for use in experiments*.* All larval injections were performed in the last pro-leg using a 0.33 mm Micro-Fine needle (BD, UK). Groups of 10 larvae were infected with 6 × 10^3^ conidia. Control groups of larvae were included in each experiment; 10 unmanipulated larvae and 10 larvae injected with 10 µl PBS. Larvae were incubated at 37 °C for 6 d. Larval death was characterised by lack of movement and melanisation (Gomez-Lopez et al., 2014, Renwick et al., 2006, Slater et al., 2011). Virulence assays were performed in duplicate.

### 2.7 Statistical analysis

Statistical significance was assessed using a two-tailed Students T-test. Survival curves comparisons were performed using a log-rank Mantel-Cox test. A p value of <0.05 was considered significant.

## 3 Results

### 3.1 Initial characterisation of the A. Fumigatus isolates

#### 3.1.1 Validation of genetic relatedness

Microsatellite typing was performed in order to verify genetic relatedness between the 13 isolates (de Valk et al., 2005). STRAf loci 3A, 3B, 3C, 4A, 4B and 4C were assessed. All isolates showed identical repeat numbers at all loci except for 3C and 4A. Isolates showed 26 repeats at 3A, 9 repeats at 3B, 12 repeats at 4B and 8 repeats at 4C. At locus 3C isolates showed 16 repeats, with the exception of isolates V130-15 and V130-18 which showed 17 repeats. At locus 4A isolates showed 9 repeats, with the exception of isolates V157-39, V157-40 and V157-80 which showed 8 repeats. As repeat numbers at these loci differed by only one single repeat the isolates are considered isogenic.

#### 3.1.2 Development of triazole resistance

According to the EUCAST clinical resistance breakpoints, isolates V130-15 and V130-14 were azole susceptible, while isolate V130-54 was itraconazole (ITR) resistant. Furthermore, isolates V130-18, V157-62, V157-59, V157-60 and V157-61 were pan-azole resistant, whereas isolates V157-39, V157-40, V157-47, V157-48 and V157-80 were ITR and posaconazole (POS) resistant (Table 1).

**Table 1:** Minimum inhibitory concentrations of the *A. fumigatus* isolates in the series.

Isolation date	Strain	Cyp51A SNP	Minimum inhibitory concentration (MIC; mg/L)		
			Itraconazole	Voriconazole	Posaconazole
22/11/11	V130-15		1	1	0.25
25/11/11	V130-14		1	1	0.25
25/11/11	V130-18		**4**	**4**	**0.5**
25/11/11	V130-54		**>16**	1	0.125
09/12/13	V157-39	**G54R**	**>16**	1	**>16**
09/12/13	V157-40	**G54V**	**>16**	1	**>16**
09/12/13	V157-47	**P216L**	**>16**	2	**>16**
09/12/13	V157-48	**P216L**	**>16**	2	**>16**
09/12/13	V157-62	**M220R**	**>16**	**8**	**>16**
12/12/13	V157-59	**M220R**	**>16**	**4**	**>16**
12/12/13	V157-60	**M220R**	**>16**	**4**	**>16**
12/12/13	V157-61	**M220R**	**>16**	**4**	**>16**
19/12/13	V157-80	**P216L**	**>16**	1	**>16**

Bold indicates a MIC exceeding the EUCAST clinical resistance breakpoint; which are defined as itraconazole >2 mg/L, voriconazole >2 mg/L and posaconazole >0.25 mg/L.

#### 3.1.3 Differences in colony morphology

**Fig. 1:**
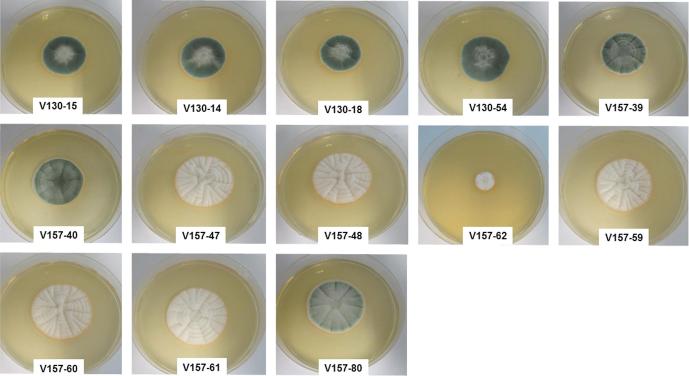
Observed colony morphology of the series. Sabouraud dextrose agar plates were spot inoculated with 5 × 10^2^ conidia and incubated at 37 °C for 96 h.

Colony morphology differed hugely between isolates. The first 5 isolates (V130-15, V130-14, V130-18, V130-54, V157-39 and V157-40) produced wild-type green colonies whereas subsequent isolates produced predominantly white, sterile hyphae. Remarkably, the final isolate collected (V157-80) produced green^1^ colonies again (Fig. 1).

### 3.2 In depth characterisation of the isolates

#### 3.2.1 Whole genome comparisons between isolates

**Fig. 2:**
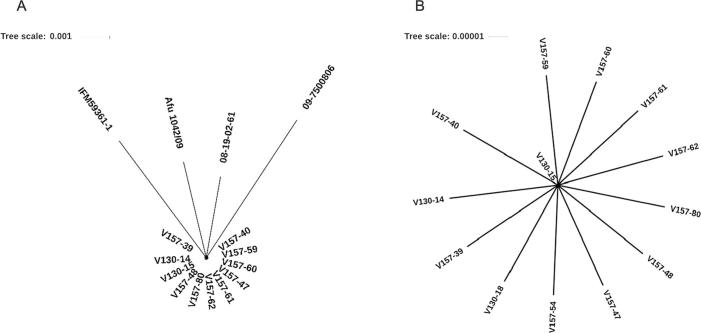
Phylogenetic tree based on whole genome sequences of the *A. fumigatus* series. (A) Single nucleotide polymorphism based phylogenetic tree was constructed using the SNPhylo pipeline and the whole genome sequences of the entire series as well as unrelated isolates IFM59361-1, 09-7500806, 08-19-02-61 and Afu 1042/09. (B) Unrooted phylogenetic tree of the series constructed using the SNPhylo pipeline. Tree scale represents nucleotide substitutions per site.

Af293 was used as the reference genome based on assessment of mapping quality and coverage statistics; mean coverage across the series was 65X and mean mapping quality was 40 (Table A.1). SNP-based full genome phylogenetic analysis was performed (Lee et al., 2014). The sequences of various unrelated isolates were included in the phylogenetic analysis; a clinical isolate from Japan (IFM59361-1) (Hagiwara et al., 2014), a clinical isolate from the UK (09-7500806) (Abdolrasouli et al., 2015), an environmental isolate from the Netherlands (08-19-02-61) (Abdolrasouli et al., 2015) and a clinical isolate from India (Afu 1042/09) (Abdolrasouli et al., 2015). Fig. 2A highlights the genetic closeness of the series, as unrelated isolates showed greater genetic distance and diversity. In agreement with microsatellite typing results, whole genome sequencing verified the isolates to be isogenic (Fig. 2A). The first strain to be isolated (V130-15) was verified to be the precursor to the series (Fig. 2B). Later isolates appear to take on independent lineages of microevolution from the precursor isolate.

A total of 248 non-synonymous SNPs, absent in early isolates V130-15 and V130-14, were identified in later isolates (Table A.2). These SNPs are predicted to have developed during the course of infection and in-host microevolution of the precursor strain (V130-15). SNPs of particular interest were identified based on their occurrence in multiple isolates or being localised in genes encoding proteins considered to play an important role in fungal cell metabolism and growth (Table 2). The genes identified were located across all 8 chromosomes and encoded a range of proteins including phospholipases (AFUA_3G07940 phosphoinositide phospholipase C), protein kinases (AFUA_2G01700 Snf1) and cell division control proteins (AFUA_3G10250 Cdc15). Cyp51A SNPs were identified in 9 isolates in the series. V157-39 harboured G54R; V157-40 harboured G54V; isolates V157-47, V157-48 and V157-80 harboured P216L; isolates V157-59, V157-60, V157-62 and V157-61 harboured M220R. SNPs were also identified in a range of uncharacterised proteins. For example, V204A SNP in AFUA_4G08100 was demonstrated in isolates V157-39, V157-48, V157-59, V157-60, V157-62, V157-61 and V157-80. A wide range of Cyp51A SNPs were identified, supporting the prediction that the isolates took on independent lineages of microevolution (Fig. 2B).

**Table 2:** Non synonymous single nucleotide polymorphisms of particular interest present in isolates within the series.

Gene	Description	V130-18	V130-54	V157-39	V157-40	V157-47	V157-48	V157-59	V157-60	V157-62	V157-61	V157-80
AFUA_1G09270	Transmembrane glycoprotein						S772N	S772N	S772N	S772N	S772N	S772N
AFUA_1G12540	TMEM1 family protein							F879C	F879C	F879C	F879C	
AFUA_2G01700	Carbon catabolite Derepressing protein kinase Snf1							R188Q	R188Q	R188Q	R188Q	
AFUA_2G02320	Hsp70 chaperone (BiP)								490^*^		490^*^	
AFUA_2G08040	C6 finger domain protein			D347Y			D347Y	D347Y	D347Y	D347Y	D347Y	D347Y
AFUA_3G07940	Phosphoinositide phospholipase C							KSS213KS	KSS213KS	KSS213KS	KSS213KS	
AFUA_3G08990	Cell surface protein	F141S	F141S									
AFUA_3G10250	Cell division control protein (Cdc15)			D220G	D220G		D220G	D220G	D220G	D220G	D220G	D220G
AFUA_3G11940	Chromatin modification-related protein			154X				292^*^	292^*^	292^*^	292^*^	154X
AFUA_4G06890	Cyp51A			G54R	G54V	P216L	P216L	M220R	M220R	M220R	M220R	P216L
AFUA_4G08100	Uncharacterised protein			V204A			V204A	V204A	V204A	V204A	V204A	V204A
AFUA_4G13800	Exo-alpha-sialidase							T214K	T214K	T214K	T214K	
AFUA_4G14310	Uncharacterised protein				A207I V209I				A207I V209I	A207I V209I	A207I V209I	A207I V209I
AFUA_5G03760	Endochitinase A1	S438P	S438P V436I									
AFUA_5G04050	Scramblase family protein			128^*^				128^*^	128^*^	128^*^	128^*^	128^*^
AFUA_6G00530	Uncharacterised protein						Y380H	Y380H	Y380H	Y380H	Y380H	Y380H
AFUA_6G10050	Small oligopeptide transporter, OPT family			G428S	G428S		G428S	G428S	G428S	G428S	G428S	G428S
AFUA_6G10620	Nuclear pore complex subunit			D43N	D43N		D43N	D43N	D43N	D43N	D43N	D43N
AFUA_6G14720	Telomere-associated RecQ helicase	I1077V	I1077V P1152L R984C	G1119R	R984C A994V S996N			D123N			T969A	

Isolates V130-15 and V130-14 have been excluded from this table as they were defined as references, and therefore did not contain any substitutions.

Of the SNPs identified, of particular interest was F125L in ZrfC alkaline zinc transporter (AFUA_4G09560) in the last isolate of the series, V157-80. F125L lies in the highly conserved ETFCND motif, which is C-terminal of a zinc-binding motif (Macpherson et al., 2005). The strict conservation of this motif indicates importance (Macpherson et al., 2005). Zinc depletion assays were performed in order to assess whether this isolate possessed any growth differences as a result of this SNP in zinc-limiting environments, much like the human host.

**Fig. 3:**
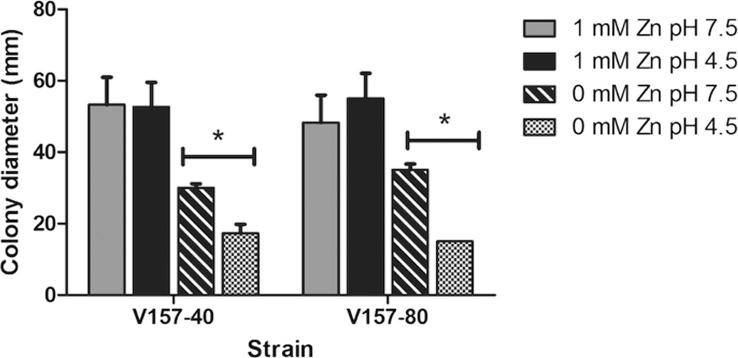
Comparison of mycelial growth of *A. fumigatus* isolates V157-40 and V157-80 in the presence and absence of zinc at pH 4.5 and 7.5. Isolates were pre-cultured on glucose minimal media lacking zinc for 7 d at 37 °C. Conidia were harvested via immersion in 30 mL PBS containing 0.05% Tween-80 and counted. Glucose minimal media plates lacking zinc or containing 1 mM zinc at pH 4.5 and 7.5 were spot inoculated with 5 × 10^2^ conidia. Every 24 h for 96 h colony diameter was measured; results at 96 h are shown. Data was obtained in triplicate and mean values ± SD are shown (^*^p < 0.05; two-tailed Students T-test).

Isolate V157-40 was selected as a control strain with a similar genetic background; both V157-40 and V157-80 possessed MICs > 16 mg/L for both ITR and POS. There were no significant growth differences between isolates V157-40 and V157-80 in the presence and absence of 1 mM zinc at pH 4.5 and 7.5. Both isolates exhibited significantly enhanced colony growth in the absence of zinc at pH 7.5 in comparison to pH 4.5 (p < 0.05). In the presence of zinc, growth differences between pH 4.5 and 7.5 were negligible in both isolates (Fig. 3). In summary, the SNP observed in ZrfC does not appear affect the ability to scavenge zinc under the conditions tested.

#### 3.2.2 Phenotypic analysis

**Fig. 4:**
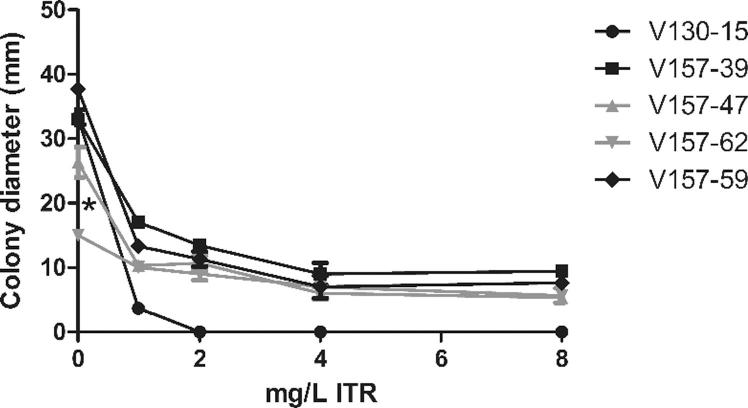
Comparison of mycelial growth of selected *A. fumigatus* isolates on solid media with increasing concentrations of itraconazole. Sabouraud dextrose agar plates were spot inoculated with 5 × 10^2^ conidia and incubated at 37 °C. Colony diameter was measured every 24 h for 96 h; results for 96 h are shown. Data was obtained in triplicate and mean values ± SD are shown (^*^p = 0.003 compared to mean of V130-15, V157-39, V157-47 and V157-59; two-tailed Students T-test).

**Fig. 5:**
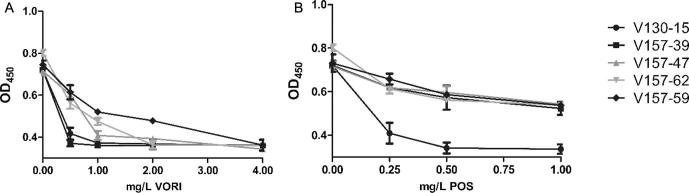
Growth kinetics of selected *A. fumigatus* isolates in liquid media. Flat-bottomed 96-well plates were seeded with 1 × 10^5^ conidia in RPMI with or without voriconazole (A) or posaconazole (B) in various concentrations. Plates were incubated at 37 °C for 48 h inside a spectrophotometric plate reader; the optical density at 450 nm was automatically measured every 20 min with 5 s shaking before every reading. Optical density at 48 h is shown. Data was obtained in duplicate, mean values ± SD are shown. No significant differences were observed between isolates under the same condition.

The isolates showed variations in their growth kinetics in control liquid and solid media. The mean OD_450_ after 48 h growth in liquid media ranged from 0.666 (V130-18) to 0.818 (V157-48) (p = NS). All isolates cultured on solid media for 96 h showed growth colony diameters of between 30.2 (V130-14) and 37.5 mm (V157-39) (p = NS). Isolate V157-62 was an exception to this range. This isolate possessed a significantly decreased mean 96 h colony diameter of 16.2 mm (p = 0.003 compared to the mean of isolates V130-15, V157-39, V157-47 and V157-59). This represents a mycelial growth rate 52% slower than the other isolates (Fig. 1, Fig. 4). Specific isolates (V130-15, V157-39, V157-47, V157-62 and V157-59) were selected for detailed phenotypic analyses to assess their response to antifungal stressors. These isolates were considered representative of the different azole resistance profiles, Cyp51A mutations and growth rates observed in the series. As shown in Table 1, V130-15 was azole susceptible without Cyp51A SNPs; V157-39 harboured G54R in Cyp51A and was ITR and POS resistant; V157-47 harboured P216L in Cyp51A and was ITR and POS resistant; V157-62 had a growth defect, harboured M220R in Cyp51A and was ITR, VORI and POS resistant; V157-59 harboured M220R in Cyp51A and was ITR, VORI and POS resistant. As anticipated, in both solid and liquid media, isolates were unable to grow in the presence of a mould-active azole at a concentration higher than its MIC. Resistant isolates exhibited a concentration dependent decrease in growth in the presence of azole antifungal agents in both solid and liquid media (Fig. 4, Fig. 5). None of the isolates exhibited enhanced growth in comparison to control conditions in the presence of azole antifungals.

The series of strains were isolated from a CGD patient. This group of patients possess a defect in the nicotinamide adenine dinucleotide phosphate (NAPDH) oxidase complex, this results in a failure to mount a phagocyte respiratory burst and produce superoxide (King et al., 2016). In order to assess whether the isolates displayed enhanced sensitivity to oxidative stress, as a result of adaptation in the CGD host, growth in the presence of *t*BOOH was assessed in both liquid and on solid media. Growth of all 5 isolates was fully inhibited by the presence of 2.5 mM tBOOH (data not shown). All 5 isolates grew normally at lower concentrations, indicating normal sensitivity to oxidative stress (Emri et al., 2015).

**Fig. 6:**
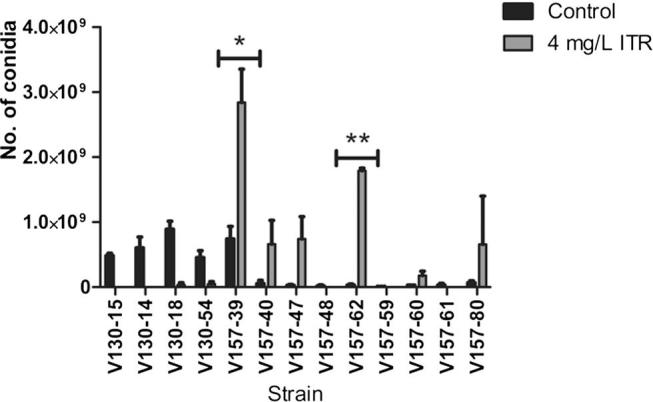
Comparison of amount of conidia produced by the *A. fumigatus* strains throughout the series. T75 culture flasks containing Sabouraud dextrose agar with or without the addition of 4 mg/L itraconazole, were inoculated with 1x10^5^ conidia and incubated at 37 °C for 7 d. Conidial suspensions were prepared *via* immersion in 30 mL PBS containing 0.05% Tween-80 and counted. Data was obtained in duplicate and mean values ± SD are shown (^*^p = 0.023, ^**^p = 0.0009; two-tailed Students T-test).

In order to further investigate the growth and development of the isolates, conidiation levels were quantified (Fig. 6). Under control conditions, specific resistant isolates (V157-39, V157-40, V157-47, V157-48, V157-62, V157-59, V157-60, V157-61, and V157-80) produced on average 5-fold less conidia than earlier isolates (V130-15, V130-14, V157-18 and V157-54) (p = 0.038). Conidia production in ITR resistant isolates (V157-39, V157-40, V157-47, V157-62, V157-60 and V157-80) increased on average 7-fold in the presence of 4 mg/L ITR (p = 0.041). Isolates V157-39 and V157-62 produced 5-fold (p = 0.023) and 3-fold (p = 0.0009) more conidia respectively in the presence of 4 mg/L ITR than susceptible isolates under control conditions. In summary, the majority of resistant isolates displayed increased levels of conidiation in the presence of 4 mg/L ITR. Isolates V157-48, V157-59 and V157-61 did not display this trend. However, upon passaging sterile hyphae onto 4 mg/L ITR, enhanced sporulation was observed.

#### 3.2.3 Differences in virulence between isolates

**Fig. 7:**
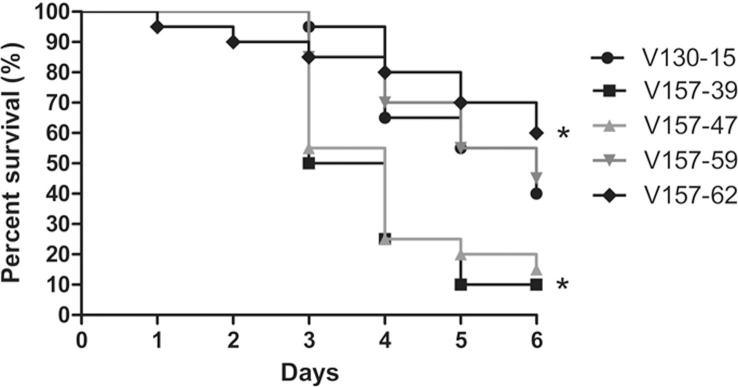
Survival of *Galleria mellonella* larvae infected with specific isolates. Groups of 10 *G. mellonella* larvae were infected with 6 × 10^3^ conidia in the last pro-leg using a 0.33 mm Micro-Fine needle. Two control groups of larvae were included in each experiment; 10 unmanipulated larvae and 10 larvae injected with phosphate-buffered saline. Larvae were monitored for 6 d; larval death was characterised by lack of movement and melanisation. Survival after infection with isolate V157-62 or V135-39 was significantly higher in comparison to isolate V130-15 (^*^p < 0.05; two-tailed Students T-test).

In order to assess associated changes in virulence in the series, survival studies were performed using the well-established invertebrate model of systemic infection, *Galleria mellonella* (Gomez-Lopez et al., 2014, Renwick et al., 2006, Slater et al., 2011). Clear differences in mortality rates were observed for the isolates tested (Fig. 7). The percentage survival at 6 d ranged from 10% (isolate V157-39) to 60% (isolate V157-62). The percentage survival at 6 d was 40% after infection with the first isolate (V130-15). Survival after infection with isolate V157-62, the only isolate with a growth defect, was significantly higher than after infection with isolate V130-15 (p = 0.037). Survival after infection with isolate V157-39 was significantly lower than after infection with isolate V130-15 (p = 0.01). No associations between virulence and conditions levels and/or resistance profile could be made.

## 4 Discussion

In this study we investigated the dynamics of both physiological and genetic adaptation in *A. fumigatus* throughout chronic and recurrent infection. Central to this study was our series of 13 isolates obtained from a single chronic granulomatous disease patient over a period of 2 years. Using this unique series, we identified large numbers of genetic changes thought to have occurred throughout infection and disease. These non-synonymous mutations identified have potential to play a role in adaptation to the human host under antifungal therapy. Additionally, we identified one isolate that displays a severe growth defect. We also observed significant differences in the ability of the isolates to produce conidia. Isolates were demonstrated to exhibit varying levels of virulence and be more equipped to cope with zinc depletion at pH 7.5 in comparison to pH 4.5, which is consistent with previous findings (Amich et al., 2010).

It is hypothesised that advantageous SNPs, which have developed during natural random mutation, are selected for during exposure to in-host stressors, such as azoles and effectors of the innate immunity. Subsequent natural selection is thought to enable survival. Here, we identified 248 SNPs predicted to have arisen during the course of infection in one host. Specific proteins were mutated in multiple isolates in the series. The identified proteins are involved in a wide range of cellular activities, indicating the stressors present in-host to be equally wide ranging. It is expected that the identified proteins play a role in in-host adaptation. Azole target Cyp51A is a hotspot for mutations conferring azole resistance (Zoll et al., 2016). Nine isolates within our series contained a SNP in Cyp51A. As the initial susceptible isolates lacked Cyp51A SNPs it can be concluded that these SNPs developed in-host, presumably as a result of azole pressure. Isolates contained either G54R, G54V, P216L or M220R SNPs, all of which have previously been proven to result in azole resistance to varying degrees (Bellete et al., 2010, Bueid et al., 2010, Chen et al., 2005, Garcia-Effron et al., 2005, Hodiamont et al., 2009, Howard et al., 2009, Kuipers et al., 2011, Mann et al., 2003, Mellado et al., 2003, Mellado et al., 2007, Mellado et al., 2004, Snelders et al., 2010, Xiao et al., 2004). In most cases the Cyp51A SNP present did not fully explain the resistance profile observed, indicating the presence of additional non-*cyp51A* mediated resistance mechanisms. As an example, isolate V157-39 was highly resistant to both POS and ITR. This isolate possessed a G54R substitution in Cyp51A, which has previously been shown to confer ITR resistance (Mellado et al., 2003). The POS resistance of this isolate is as yet unexplained.

Four isolates possessed SNP R188Q in Snf1 kinase; this protein is known to be involved in nutrient limitation and salt stress responses in *Saccharomyces cerevisiae* (Hsu et al., 2015, Sanz, 2003). It is possible that R188Q alters Snf1 functionality, potentially enhancing the ability of the isolates to cope with these stresses, which could enable persistence of infection. Furthermore, 7 isolates possessed SNP D347Y in C6 finger domain protein (AFUA_2G08040). This protein possesses RNA polymerase II transcription factor activity and is zinc ion binding (Bateman et al., 2015). Interestingly, Hagiwara *et al* also reported a mutation (Y958) in this C6 finger domain protein, predicted to have developed throughout infection in an invasive pulmonary aspergillosis patient (Hagiwara et al., 2014). This supports our hypothesis that the identified proteins are involved in in-host adaptation.

Fitness losses in clinical azole resistant *A. fumigatus* isolates are frequently reported (Hagiwara et al., 2014, Valsecchi et al., 2015). Various methods of assessing fitness are described in *A. fumigatus* (Arendrup et al., 2010, Lackner et al., 2017, Valsecchi et al., 2015)*.* In this study we performed liquid and solid media growth assays. These growth assays were selected to represent different forms of *in vivo* growth. Liquid assays were deemed a basic representation of growth in human tissue, where conidiation does not occur. Solid media assays were chosen to crudely represent growth with sporulation, which occurs when the fungus is in contact with the air, as on the epithelial lining of the airways. Isolate V157-62 was shown to harbour defects in both conidiation and growth on solid media. This isolate contains a M220R Cyp51A mutation. Previous *in vivo* competition studies, using both immunocompetent and immunosuppressed mice, have shown that M220 SNPs are not associated with fitness costs (Lackner et al., 2017, Valsecchi et al., 2015). It is possible that other SNPs gained as a result of adaptation are the cause of this fitness defect. These SNPs could be either beneficial and associated with a fitness cost or simply disadvantageous. Clues can be obtained using whole genome sequencing however further research is required to definitively associate specific SNPs, or combinations thereof, with this phenotype.

Later isolates were shown to produce significantly fewer conidia than earlier more susceptible isolates. Conidiation in specific isolates was restored in the presence of itraconazole. This could be classified as azole addiction, where the fungus has adapted to grow in the presence of azole and as a result requires it for specific aspects of growth. These isolates do not share an isolation date or resistance profile, but perhaps shared localisation within the lung and therefore adapted similarly. As the majority of isolates possessed normal mycelial growth rates, the defect lies directly in the isolates’ ability to form conidia. In agreement with our findings, Hagiwara et al also reported a sporulation defect in serially isolated clinical strains from individual patients (Hagiwara et al., 2014). It can be hypothesised that the virulence of the poorly sporulating isolates is unaffected by this defect as conidiation is rarely observed in human tissue and is not required for invasive disease pathogenesis. However, the environmental spread of resistant isolates with this defect is likely to be limited.

Interestingly, under zinc depletion isolates grew better at pH 7.5 in comparison to pH 4.5. ZrfC is central to this behaviour. This zinc transporter is capable of functioning under alkaline zinc limiting conditions due to its N-terminus, which is also predicted to scavenge Zn^2+^ from host tissues (Amich et al., 2014, Wilson et al., 2012). It is probable that these strains have evolved in host and are therefore more adapted to scavenge zinc and thrive at physiological pH in host. Survival in acidic conditions is perhaps driven by adaptation to the ecological niche of *A. fumigatus* in soil. The SNP identified by us in the ZrfC in V157-80 did not influence the capability to grow in alkaline zinc limiting conditions and seems not to play a role in in-host adaptation.

The phenotypic and genotypic changes observed in the series may be associated with the virulence differences observed in our experimental *G. mellonella* model. Isolates exhibited both increased and decreased virulence in comparison to the precursor isolate (V130-15). The isolate determined to have a growth defect *in vitro* (V157-62) showed attenuated virulence in comparison to V130-15. This could be a direct impact of its slower mycelial growth. Another azole resistant isolate (V157-39) showed enhanced virulence in comparison to V130-15. In agreement with previous findings, no associations could be made between resistance development and changes in virulence (Lackner et al., 2017, Valsecchi et al., 2015). Furthermore, no associations could be made between conidiation levels and virulence. As mycelial growth drives invasion in-host rather than conidiation, it is possible that differences in conidiation ability have minimal impact on ability to cause infection. It is likely that microevolution has driven both increases and decreases in virulence. Attenuated virulence may well be a cost associated with another yet unidentified adaptation mechanism. Increases in virulence are regarded as direct adaptation to enable persistence.

## 5 Conclusions

In summary, *A. fumigatus* undergoes substantial in-host adaptation. This adaptation occurs on both a physiological and genetic level as illustrated by our results, and is hypothesised to enable persistence of infection in some cases. Genetic changes reported here are wide ranging, suggesting that the stressors driving adaptation are equally wide ranging. It should be noted that as this study involves a series of isolates from a single chronic granulomatous disease patient, adaptation dynamics reported may not be representative of other patient groups and/or other patients with chronic granulomatous disease. However, this study is the first to provide in depth analysis into the genetic and physiological changes that occur in *A. fumigatus* during adaptation to the human host.

## Figures and Tables

**Table A1:** Mean coverage and mapping quality of whole genome sequence data sets to Af293 reference.

Strain	Mean coverage (X)	Mean mapping quality
V130-15	69	40
V130-14	85	39
V130-18	118	39
V130-54	69	39
V157-39	62	40
V157-40	76	41
V157-47	20	37
V157-48	25	37
V157-62	53	41
V157-59	77	40
V157-60	58	41
V157-61	61	39
V157-80	71	41

Mean across series	65	40

**Table A2:** Non synonymous single nucleotide polymorphisms identified to have developed throughout the course of infection in the series of isolates.

Gene	Description	V130-18	V130-54	V157-39	V157-40	V157-47	V157-48	V157-59	V157-60	V157-62	V157-61	V157-80
AFUA_1G00220	Uncharacterised protein										A430V T437I 348 S493L	
AFUA_1G00230	Uncharacterised protein				748^*^		L756A		748^*^			
AFUA_1G00550	Uncharacterised protein	A430V			T11M		V369L			S168L M170I V173I D175N R176C T182C T182I		
AFUA_1G00750	Uncharacterised protein							171 A170T M162I P155L M153I S152L				
AFUA_1G12130	Translation initiation factor eIF4E							V160A				
AFUA_1G06060	Uncharacterised protein			A786T G608V S772N								
AFUA_1G08920	IQ calmodulin-binding motif domain protein						A189V					
AFUA_1G09270	Transmembrane glycoprotein						S772N	S772N	S772N	S772N	S772N	S772N
AFUA_1G12540	TMEM1 family protein							F879C	F879C	F879C	F879C	
AFUA_1G15940	Auxin Efflux Carrier superfamily						G318GGGS				G281C	
AFUA_2G00230	Amid-like NADH oxidoreductase			E240A								
AFUA_2G01700	Carbon catabolite derepressing protein kinase Snf1							R188Q	R188Q	R188Q	R188Q	
AFUA_2G02320	Hsp70 chaperone (BiP)								490^*^		490^*^	
AFUA_2G04290	Cytochrome P450 monooxygenase				N176S							
AFUA_2G07450	PX domain protein										L282F	
AFUA_2G08040	C6 finger domain protein			D347Y			D347Y	D347Y	D347Y	D347Y	D347Y	D347Y
AFUA_2G09710	Protein kinase (NpkA)											P414L
AFUA_2G11570	F-box domain protein			G204V								
AFUA_2G12360	WD repeat protein				E942K							
AFUA_2G13500	Uncharacterised protein							T				
AFUA_2G13770	C2H2 conidiation transcription factor FlbC								218^*^			
AFUA_2G16170	DNA-directed RNA polymerase III RPC4		R16A									
AFUA_2G13960	Tafazzin				L157F							
AFUA_2G16830	Endonuclease/exonuclease/phosphatase family protein									G190R		
AFUA_2G17000	PT repeat family protein	E1362K										
AFUA_2G17600	Conidial pigment polyketide synthase PksP/Alb1				S1197Y							
AFUA_2G18100	Telomere-associated RecQ helicase											
AFUA_3G02550	Uncharacterised protein										Y33F S34L	
AFUA_3G02640	Nucleoside-diphosphate-sugar epimerase family protein										237 A243T T248C	
AFUA_3G03852	C2H2 type zinc finger domain protein				C292Y							
AFUA_3G04300	Actin cytoskeleton organization and biogenesis protein				P1125H							
AFUA_3G07080	UPF0016 domain protein				V470M							
AFUA_3G07940	Phosphoinositide phospholipase C							KSS213KS	KSS213KS	KSS213KS	KSS213KS	
AFUA_3G08570	Uncharacterised protein											V54L
AFUA_3G08990	Cell surface protein	F141S	F141S									
AFUA_3G10250	Cell division control protein (Cdc15)			D220G	D220G		D220G	D220G	D220G	D220G	D220G	D220G
AFUA_3G10290	Uncharacterised protein									R198W		
AFUA_3G10450	Uncharacterised protein				R718C							
AFUA_3G11940	Chromatin modification-related protein			154X				292^*^	292^*^	292^*^	292^*^	154X
AFUA_3G14445	Bromodomain associated domain protein				R223C							
AFUA_3G14510	Rhamnogalacturonan acetylesterase RgaE									P186L		
AFUA_3G14700	Polyketide synthase										A1923G	
AFUA_4G04760	Inositol kinase kinase (UvsB)			E485Q								
AFUA_4G06890	Cyp51A			G54R	G54V	P216L	P216L	M220R	M220R	M220R	M220R	P216L
AFUA_4G07530	Uncharacterised protein						288HDD					
AFUA_4G08100	Uncharacterised protein			V204A			V204A	V204A	V204A	V204A	V204A	V204A
AFUA_4G08360	Mediator of RNA polymerase II transcription subunit 8		E188V									
AFUA_4G09560	ZIP Zinc transporter											F125L
AFUA_4G12050	Thermoresistant gluconokinase family protein			L196V								
AFUA_5G12440	Cell cycle control protein (Cwf23)					A242V						
AFUA_4G13000	Lysine-specific histone demethylase Aof2				E2D							
AFUA_4G13800	Exo-alpha-sialidase							T214K	T214K	T214K	T214K	
AFUA_4G14310	Uncharacterised protein				A207I V209I				A207I V209I	A207I V209I	A207I V209I	A207I V209I
AFUA_5G02390	Auxin Efflux Carrier superfamily				V403D							
AFUA_5G03760	Endochitinase A1	S438P	S438P V436I									
AFUA_5G04050	Scramblase family protein			128^*^				128^*^	128^*^	128^*^	128^*^	128^*^
AFUA_5G07140	Translation elongation factor G2				A542S							
AFUA_5G08330	RNA binding protein											T209S
AFUA_5G08390	Response regulator			V706F								
AFUA_5G11820	Survival factor 1									213^*^		
AFUA_5G14920	Uncharacterised protein	W372S										
AFUA_5G14865	Uncharacterised protein										301^*^	
AFUA_6G00530	Uncharacterised protein						Y380H	Y380H	Y380H	Y380H	Y380H	Y380H
AFUA_6G04080	Uncharacterised protein				A1393T							
AFUA_6G05280	Meiosis protein MEI2				K417N							
AFUA_6G06335	Histone-lysine N-methyltransferase, H3 lysine-4 specific			R578C								
AFUA_6G07660	Uncharacterised protein										E169K	
AFUA_6G10050	Small oligopeptide transporter, OPT family			G428S	G428S		G428S	G428S	G428S	G428S	G428S	G428S
AFUA_6G10380	Cullin binding protein CanA									L91V		
AFUA_6G10490	Uncharacterised protein											Y365C
AFUA_6G10620	Nuclear pore complex subunit			D43N	D43N		D43N	D43N	D43N	D43N	D43N	D43N
AFUA_6G12950	Alpha, alpha-trehalose-phosphate synthase (UDP-forming)									V265A		
AFUA_6G13660	Uncharacterised protein										P865L	
AFUA_6G13800	Integral membrane protein Pth11-like								W148C			
AFUA_6G13900	Uncharacterised protein											K413N
AFUA_6G14720	Telomere-associated RecQ helicase	I1077V	I1077V P1152L R984C	G1119R	R984C A994V S996N			D123N			T969A	
AFUA_7G00720	Uncharacterised protein	A311T					A311T				A311T	
AFUA_7G01290	Uncharacterised protein					G51R						
AFUA_7G01310	C6 transcription factor											Q538H
AFUA_7G01960	Uncharacterised protein									167^*^		
AFUA_7G04180	Amine oxidase				R614P							
AFUA_7G05220	Mitochondrial carrier protein											V639L
AFUA_7G05960	C2H2 finger domain protein				G792D							
AFUA_7G08250	C6 finger domain protein	S749G							S749G			
AFUA_7G08630	Uncharacterised protein											
AFUA_8G00750	C2H2 transcription factor	Q429K		C45Y								
AFUA_8G01820	Uncharacterised protein						GX					
AFUA_8G02420	Uncharacterised protein				E							
AFUA_8G02850	Actin binding protein											S505T
AFUA_8G04520	Cytoskeleton assembly control protein Sla1			Q257K								
AFUA_8G05780	NACHT and Ankyrin domain protein					K618E Q622H						
AFUA_8G06132	FluG domain protein	G81R M90I A135I E142K R79H T82M	A135I									
AFUA_8G06140	Sensor histidine kinase/response regulator	A231I F232L M222D S223N T218A										
AFUA_8G06210	Metalloreductase transmembrane component					R479Y						
AFUA_8G06220	Uncharacterised protein			S238L V245I								S238L 239 V245I 249 V249M G252S 253
AFUA_8G06230	Uncharacterised protein				V50M							

Isolates V130-15 and V130-14 have been excluded from this table as they were defined as references, and therefore did not contain any substitutions.

## Footnotes

1 For interpretation of color in Fig. 1, the reader is referred to the web version of this article.
